# The International Congress on Alcoholism

**Published:** 1909-07-24

**Authors:** 


### THE INTERNATIONAL CONGRESS ON ALCOHOLISM.

The twelfth International Congress on Alcoholism, which we announced some few weeks ago, is now in progress as we go to press with this issue of The Hospital. In view of the prominence which we have consistently given in these columns to the whole question of alcohol in its many and varied aspects—moral, sociological, pathological, dietetic and therapeutic—and the recent publication by us of the third of our Commission Reports on alcoholic beverages, it is natural that we should now watch with much interest and close attention the proceedings of the present congress in London. Moderation and scientific accuracy are essential to the success of any such large and elaborate attempt to accumulate evidence and report progress upon a matter of vital importance to the welfare of the race. Public opinion, which is the only permanent medium for affecting reform, is nowadays suspicious of any doctrine of social and moral regeneration whose chosen or self-selected advocates adopt a narrow, partisan and intolerant attitude. Those who, in pressing their views and proposals upon the community, "protest too much," and allow no measure of right or of honesty to their opponents, are only too often the means of stultifying the efforts of their equally sincere but less fanatical associates.

In that field of humanitarian reform, which concerns the protection of the animal kingdom from the oppression, ignorance and neglect of mankind, the Society for the Prevention of Cruelty to Animals has succeeded in its admirable work, and has indeed revolutionised the attitude of the lower and baser section of society towards dumb animals, because of the sanity, simplicity and moderation of its line of action. By keeping steadfastly aloof from all those hot-headed inconsequent zealots, whose undisciplined outbursts only tend to obscure the real issues of the matter, and to retard progress in kindness and justice to animals, this Society has accomplished a great work, and earned the confidence and goodwill of every thinking person. So, as we take it, must the alcohol reformers do also, if they would succeed in like measure. And the holding of a responsible conference, at which, among other activities, unimpassioned scientific papers are read, and orderly practical discussions follow, is a means of progress, with which we gladly profess ourselves in sympathy. Of course we do not approve of every feature of the Congress, or view with satisfaction the more lurid and extravagant specimens displayed in the associated Exhibition. But we quite realise that an international conference, which aims at co-operation between all the varied and scattered units of the anti-alcohol army, as well as at inter-communication and cohesion between the scientific investigators of alcoholism in its every aspect, must include many delegates with extreme views and methods, and many features which seem useless and retrogressive to those who think as we do. The inclusive and representative nature of the Congress must therefore, we suppose, excuse whatever in it appears grotesque or futile or even pernicious to the advocates of true temperance.

As was well said by *The Times* on Monday "this is not a congress of teetotallers only. No test of opinion has been imposed on the delegates or other members." And we may hope that among the many papers those of high scientific importance will counterbalance those which seem to prejudice the advance of social reform.

